# Interconnected Gene Networks Underpin the Clinical Overlap of *HNRNPH1*-Related and Rubinstein–Taybi Intellectual Disability Syndromes

**DOI:** 10.3389/fnins.2021.745684

**Published:** 2021-10-25

**Authors:** Lidia Larizza, Valentina Alari, Luciano Calzari, Silvia Russo

**Affiliations:** Laboratory of Medical Cytogenetics and Human Molecular Genetics, Biomedical & Technologies Research Center, Istituto di Ricerca e Cura a Carattere Scientifico (IRCCS) Istituto Auxologico Italiano, Milan, Italy

**Keywords:** Rubinstein–Taybi syndrome, iPSC neurons, transcriptome analysis, RNA binding proteins, *HNRNPH1*-related ID syndrome, clinical overlapping, shared genes network

## Introduction

The transcriptome of induced pluripotent stem cell (iPSC)-neurons (iNeurons) generated from patients with neurodevelopmental disorders (NDDs) is a quantitative phenotype that provides the biological context for unveiling the molecular pathways disrupted in the “diseased” cells. RNASeq of iNeurons from individuals with Rubinstein–Taybi syndrome (RSTS, MIM# 180849, and #613684), an intellectual disability (ID) disorder caused by monoallelic pathogenic variants in the genes encoding CBP/p300 lysine acetyltransferases (Hennekam, 2006), revealed that RSTS-univocal downregulated genes (DRGs) encoding a coherent network of RNA-binding proteins (RBPs) are implicated in RNA processing and ribosome complex biogenesis (Calzari et al., 2020). Variation or misregulation of several genes for heterogeneous nuclear ribonuclear proteins (hnRNP), a well-characterized family of RBPs implicated in RNA splicing and processing (Geuens et al., 2016), has been linked to neurological and neurodevelopmental disorders supporting a variably shared molecular pathogenesis of disease subtypes that enhances the identification of new family-related NDD genes (Gillentine et al., 2021). Interestingly, like hnRNPs, lysine acetyltransferases have been identified as a gene family involved in RNA processing reaching the FDR significance from meta-analyses of >10.000 NDD exomes (Coe et al., 2019; Gillentine et al., 2021). We discuss herein RSTS and *HNRNPH1*-related syndromic ID based on their interconnected gene networks and shared phenotypic spectra.

### Downregulated RNA-Binding Proteins for Alternative Splicing and Ribosome Biogenesis in Rubinstein–Taybi Syndrome iNeurons

**Figure 1:**
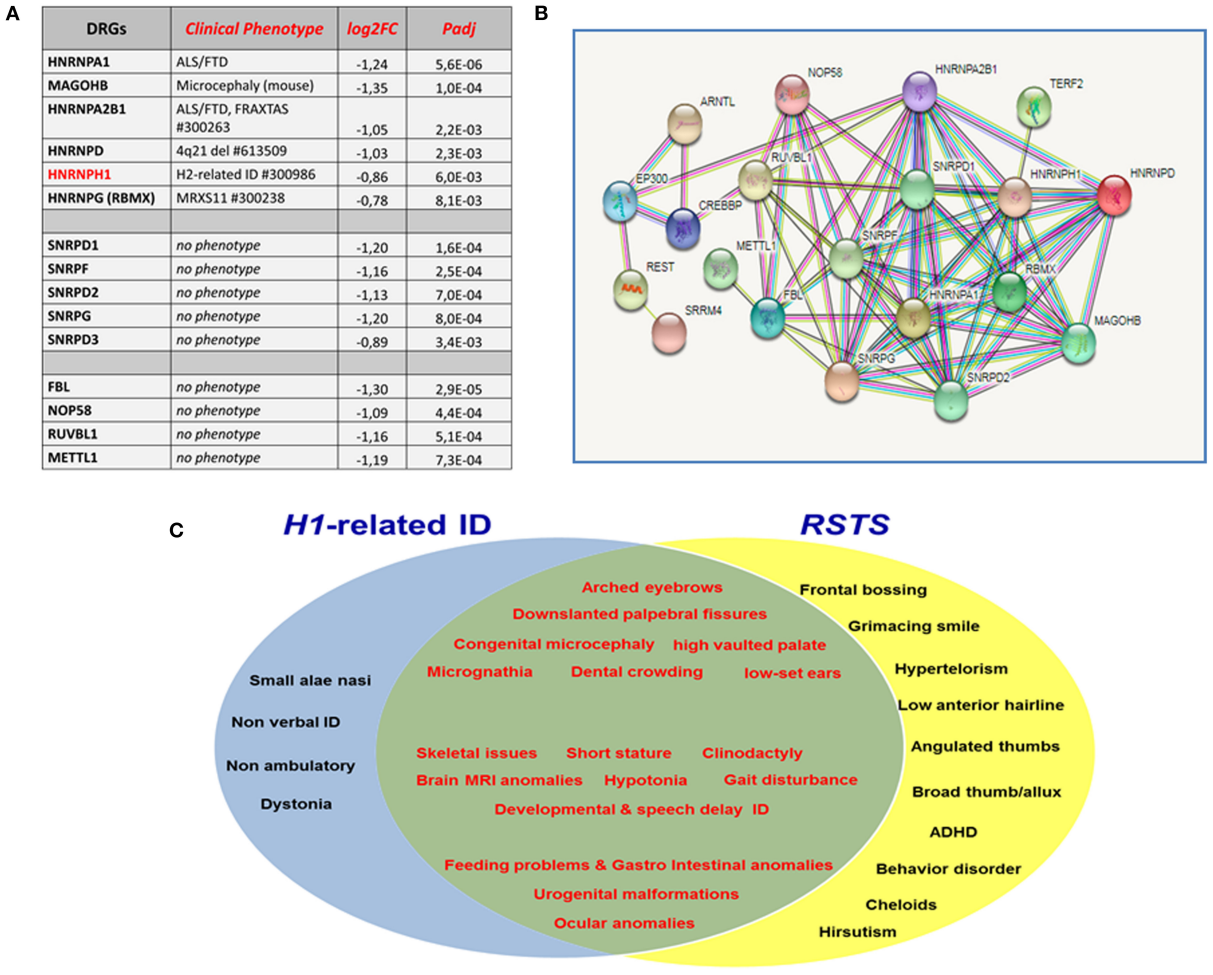
**(A)** Main groups of downregulated genes (DRGs) in RSTS iNeurons all encoding RNA-binding proteins (RBPs). A prominent set of DRGs encode heterogeneous nuclear ribonucleoproteins (hnRNP) family members, which are involved in all steps of RNA processing: their mutation/downregulation leads to the indicated neurological/neurodevelopmental disorders. Another set of DRGs encode five of the seven core proteins shared by the small nuclear ribonucleoproteins (snRNPs) of the pre-mRNA splicing machinery and also acting in pre-ribosomal RNA processing. Other DRGs encode key nucleolar/ribosomal proteins which defect leads to impaired ribosome biogenesis and disruption of the mutual interactions between RNA processing and chromatin regulation. Fold change levels (Log2FoldChange) and statistical significance values adjusted for multiple comparison (padj-False Discovery Rate) are provided for each gene. To identify differentially expressed genes [DRGs as well as URGs (upregulated genes)], a paired differential gene expression (DGE) analysis (using patients/controls as covariate) was conducted by using DESeq2 package (Love et al., 2014) in R environment, comparing the expression profile of mature neurons with neural progenitors of patients and controls, separately (iNeurons vs. neural progenitors). Genes with a padj <0.01 [Benjamini–Hochberg (false discovery rate)] were considered differentially expressed (DEGs). RSTS- and control-specific signatures were highlighted by combining/crossing RSTS and controls DEGs lists. **(B)** The multiple interactions between CBP/p300 and RNA-binding proteins in STRING database (https://string-db.org/) account for the DRGs pointed out by transcriptome analysis of *CREBBP/EP300* defective neurons and the consequent perturbed cross-talk between chromatin regulators and RBPs. **(C)** Venn diagram showing the clinical overlap of *HNRNPH1*-related and Rubinstein–Taybi syndrome (RSTS).

Out of the multiple downregulated genes specific of RSTS iNeurons, representative ones of each set, are listed, in order of increasing adjusted (false discovery rate) *p*-values, in Figure 1A. A first set encodes the hnRNP (heterogeneous nuclear ribonucleoprotein) family members hnRNPA1, MAGOHB, hnRNPA2B1, hnRNPD, hnRNPH1, and hnRNPG (alias RBMX), all involved in alternative splicing, a process which magnitude is remarkably increased in the nervous system, due to the huge number of isoforms needed for the development of neural cell type-specific properties, synapse specification, and establishment of functional networks (Furlanis and Scheiffele, 2018). Mis-splicing events have severe repercussion on neuronal functions, as attested by the recognized neurodegenerative and neurodevelopmental disorders resulting from mutation/mis-regulation of all these genes (Figure 1A). The *HNRNPH1* gene has been recently implicated in a syndromic form of intellectual disability (Pilch et al., 2018; Reichert et al., 2020), which presents some phenotypic overlap with *H2*-caused or Bain-type ID (Bain et al., 2016, 2021). Both NDDs result from monoallelic pathogenic variants preferentially affecting the nuclear localization domain, indispensable to nuclear transport of the hnRNPH1/H2proteins, though *HNRNPH1* variant types, different from *HNRNPH2* ones, span the entirety of hnRNPH1 (Gillentine et al., 2021). The main function of hnRNPH1/H2 is to control microexon inclusion and inhibition of cryptic polyadenylation sites during neurogenesis, safeguarding the integrity of the transcriptome. Both paralogs, hnRNPH1 with a stronger effect than hnRNPH2, control the splicing of differentiation factor TRF2 (telomeric repeat binding factor 2, alias TERF2), promoting the expression of the long isoform, which acts as the inhibitor of neural differentiation, as well as the splicing of other neural genes (Grammatikakis et al., 2016). DRGs for RNA-processing proteins, also involved in ribosome complex biogenesis include those for the polypeptides D1, F, D2, G, and D3 making up the structural core of small nuclear ribonucleoproteins (snRNPs) of the eukaryotic pre-mRNA splicing machinery (Figure 1A). Further representative top DRGs contributing to the weakened RBPs network in RSTS iNeurons encode the nucleolar proteins fibrillarin and NOP58, which, similar to the “dual player” proteins RUVBL1 and METTL1, act on both the transcriptome and the RBP-ome (Figure 1A): their downregulation in *CREBBP/EP300* defective iNeurons is predicted to disrupt the physiological cross-talk between chromatin and post-transcriptional regulators (Larizza et al., 2022).

### Integrating Transcriptome Data With Protein Interaction Networks and Experimental Evidence From the Literature

Figure 1B shows the overall connections between CBP/p300 and the main members of hnRNP, snRNP, and ribosomal proteins downregulated in RSTS iNeurons according to the STRING protein–protein interactions database (https://string-db.org/). Moreover, experimentally determined evidence in the literature connects both MAGOHB, core component of the exon junction complex (EJC) through its peripheral proteins and the core proteins of the uridine-rich small ribonucleoproteins (snRNPs) to the neural-specific microexon splicing program governed by the serine arginine repetitive matrix 4 (SRRM4) protein, which reduced expression in *Ep300* and *Crebbp*-depleted mouse neurons, has been linked to cognitive dysfunction and autism (Gonatopoulos-Pournatzis et al., 2018). The direct interaction between CBP/p300 and the circadian protein ARNTL (alias BMAL1) is well-supported by STRING, while that between ARNTL and NOP58 is not yet evidenced by STRING, but has been experimentally proven (Cervantes et al., 2020).

### Clinical Overlap Between *HNRNPH1*-Related and Rubinstein–Taybi Intellectual Disability Syndromes

Despite a small number of *HNRNPH1*-mutated patients that have been clinically evaluated so far (seven plus a fetus), the high sequence homology (96%) of hnRNPH1 and hnRNPH2 has immediately raised the cross-comparison of unique and overlapping phenotypic features of this new NDD with the previously described *H2*-related syndromic ID (Bain et al., 2016, 2021). Both NDDs share developmental and speech delay, ID, a few dysmorphic features, gastrointestinal anomalies, and skeletal issues, but also present distinctive signs. These are represented in *H1*-mutated patients by congenital microcephaly, arched eyebrows, downslanted palpebral fissures, long hanging columella and small alae nasi, high-vaulted palate, and a higher incidence of congenital anomalies of the brain and genitourinary and ophthalmological systems (Pilch et al., 2018; Reichert et al., 2020). Interestingly, the well-characterized Rubinstein–Taybi syndrome shows clinical signs common to both NDDs, in keeping with the assessed role of hnRNP and lysine acetyltransferases gene families as NDDs candidates (Gillentine et al., 2021), but it especially overlaps the *H1*-related clinical spectrum, as illustrated in Figure 1C. The phenotypical and gene expression evidence that *HNRNPH1* and *HNRNPH2-*caused syndromes are independent disorders (Gillentine et al., 2021) lines up with the inclusion of *HNRNPH1* and not *HNRNPH2* among the DRGs for hnRNP family members in RSTS iNeurons.

## Discussion

Data from transcriptome analysis of RSTS neurons definitely connects CBP/p300 deficit to dysfunction of a coherent network of RBPs involved in RNA processing and ribosome complex biogenesis (Calzari et al., 2020; Larizza et al., 2022). It has been suggested that disruption of a core set of hnRNP proteins sharing similar molecular structure and function and enriched expression in an actively dividing glia may lead to abnormal development (Gillentine et al., 2021).

A recent proteomic study of the mouse brain interactome has highlighted the connectivity of >1,000 multi-protein complexes including hundreds of brain-specific assemblies enriched for RNA-binding proteins and associated to core neurological processes and disorders, suggesting that disruption of complex function primed by multiple components and routes can lead to similar phenotypic outcome (Pourhaghighi et al., 2020). By using reciprocal pull-downs and a transgenic model, the authors validated a 28-member RNA-binding protein complex associated with amyotrophic lateral sclerosis, which includes hnRNPH1 (Pourhaghighi et al., 2020), in line with a study reporting on the role of hnRNPH-F sequestered together with other hnRNPs in toxic RNA foci in the spinal cord of amyotrophic sclerosis patients (Cooper-Knock et al., 2015). To note (Figure 1A), *HNRNPA1* and *HNRNPA2B1* genes, involved in ALS, are top DRGs in RSTS iNeurons.

Post-translational modifications of hnRNPs upon shuttle to the cytosol regulate their stability and temporal and spatial functions, raising the possibility that defective lysine acetylation of splicing factors besides their predicted diminished amount might contribute to impaired RNA processing in RSTS iNeurons. In keeping with the coupling of transcription to splicing both physically and kinetically (Luco et al., 2011), many chromatin regulators of alternative splicing were identified including the p300 acetyltransferase found to regulate alternative splicing by modulating binding to pre-mRNA of its target CD44 gene of the acetylated splicing factors hnRNPM and Sam68 (Siam et al., 2019). Sam68 is a KH (hnRNPK homology) domain protein, already known to be acetylated by CBP (Hong et al., 2002). Precise mapping of the modified lysine residues in splicing factors included in the vast CBP/p300 acetylome (Weinert et al., 2018) could permit the study of the magnitude of the effect on splicing resulting from CBP/p300 deficiency.

Numerous reciprocal interactions are established between hnRNPs through their C-terminus intrinsically disordered regions (IDRs), present in CBP/p300 too (Dyson and Wright, 2016), and between them and diverse RBPs with partially shared targetomes, implying that downregulation/mutation of each member of the complex has a repercussion on the others leading to subverted neuronal homeostasis and disease. Tight acetylation/deacetylation of IDRs has been proposed as a mechanism regulating liquid–liquid phase separation (LLPS) of the IDR-containing proteins, ensuring that the biogenesis of membrane-less organelle in response to stress does not pass the limit rendering RNA-binding proteins prone to misfolding and aggregation connected to neurodegenerative disease (Saito et al., 2019). Though the contribution of LLPS to pathological processes in living cells is only little explored, an altered balance between lysine acetyltransferases and deacetylases is likely negatively impacting on this process.

All these considerations may account for the striking clinical similarity of *HNRNH1*-related and RSTS ID syndromes. However, additional *HNRNPH1*-mutated individuals should be clinically evaluated to confirm their clinical resemblance to RSTS and their limited overlap with *HNRNPH2*-caused ID, to substantiate this opinion.

## Author Contributions

LL, VA, LC, and SR gave substantial contributions to the conception and design of the work. LL wrote the first draft of the manuscript. All authors approved the final and submitted version.

## Funding

This study was supported by the Italian Ministry of Health RC 08C921 to the IRCCS Istituto Auxologico Italiano.

## Conflict of Interest

The authors declare that the research was conducted in the absence of any commercial or financial relationships that could be construed as a potential conflict of interest.

## Publisher's Note

All claims expressed in this article are solely those of the authors and do not necessarily represent those of their affiliated organizations, or those of the publisher, the editors and the reviewers. Any product that may be evaluated in this article, or claim that may be made by its manufacturer, is not guaranteed or endorsed by the publisher.
